# Feasibly of axitinib as first-line therapy for advanced or metastatic renal cell carcinoma: a single-institution experience in Japan

**DOI:** 10.1186/s12894-015-0027-4

**Published:** 2015-04-16

**Authors:** Takuya Koie, Chikara Ohyama, Takahiro Yoneyama, Hayato Yamamoto, Atsushi Imai, Shingo Hatakeyama, Yasuhiro Hashimoto, Tohru Yoneyama, Yuki Tobisawa, Kazuyuki Mori

**Affiliations:** Department of Urology, Hirosaki University Graduate School of Medicine, 5 Zaifucho, Hirosaki, 036-8562 Japan

**Keywords:** Axitinib renal cell carcinoma, First-line, Vascular endothelial growth factor receptor, Advanced renal cell carcinoma, Metastatic renal cell carcinoma

## Abstract

**Background:**

Clinical benefit of axitinib as a first line agent to treat patients with metastatic renal cell carcinoma (mRCC), or locally advanced renal cell carcinoma (RCC) have not been clearly demonstrated. The aim of this study was to evaluate the efficacy and safety of axitinib as first-line therapy in Japanese patients with locally advanced RCC or mRCC.

**Methods:**

In this retrospective study, we focused on eighteen patients who underwent first-line therapy with axitinib between May 2012 and May 2014 at Hirosaki University. Axitinib was orally administered at a dose of 10 mg daily. Progression-free survival (PFS) was the primary endpoint, while secondary endpoints included overall response rate (ORR) and adverse events (AEs).

**Results:**

All patients had histologically proven clear cell RCC. The median duration of the administration of axitinib was 10.8 months. According to the response evaluation criteria for solid tumors, five patients (27.8%) achieved a partial response and nine (50%) had stable disease. The 1-year PFS rate was 84.4%, and the median PFS was 20.4 months (95% confidence interval, 17.5 – 21.7). No serious AEs were reported during the study, and there were no toxicity-related deaths.

**Conclusions:**

In the current study, axitinib showed acceptable oncological outcomes and favorable safety profile as first-line therapy for locally advanced RCC or mRCC in treatment-naïve Japanese patients. Thus, first-line therapy with axitinib may provide a feasible option for treatment of advanced RCC or mRCC patients.

## Background

Although the innate chemoresistance of renal cell carcinoma (RCC) is a limitation in the systemic treatment for metastatic renal cell carcinoma (mRCC) [1], the clinical benefits of using targeted agents to treat patients with mRCC or locally advanced RCC have become increasingly clear [2]. Currently, six targeted agents are approved for the treatment of mRCC in Japan, including the multi-targeted receptor tyrosine kinase inhibitors (TKIs): sunitinib, pazopanib, axitinib and sorafenib; and the mammalian target of rapamycin inhibitors (mTORs): everolimus and temsirolimus.

There is no doubt that initial treatment of low- or intermediate-risk mRCC patients [3] with a vascular endothelial growth factor (VEGF)-targeted agent significantly improves clinical outcomes compared with conventional immunotherapy [4]. Of these, sorafenib, sunitinib and pazopanib have been approved as first-line treatment for advanced RCC or mRCC based on several clinical trials conducted in Western countries [2-4]. Similarly, first-line therapy with temsirolimus has demonstrated efficacy in patients with poor-risk mRCC [3,5]. However, based on the incidence and severity of adverse events (AEs) in several clinical trials [6-9], Japanese patients with mRCC have been shown to exhibit greater AEs to TKIs compared with their Western counterparts.

Axitinib, an effective and selective second-generation inhibitor of VEGF receptors-1, 2, and 3 [10], has demonstrated clinical efficacy in patients with mRCC in phase II studies [11,12]. Single-agent axitinib is active and well tolerated as a second-line treatment for mRCC [11,12]. Conversely, no significant increase in progression-free survival (PFS) was found in treatment-naïve mRCC patients who were treated with axitinib, when compared with those treated with sorafenib [13]. However, it is possible that Japanese patients may exhibit a different response to axitinib, in terms of antitumor effects or profile of AEs, when compared with their Western counterparts [14]. In the National Comprehensive Cancer Network guideline 2015, axitinib is recommended as a treatment option for first-line therapy in patients with locally advanced or metastatic RCC.

This study, which was carried out at a single institution in Japan, aimed to evaluate the efficacy and safety of axitinib as first-line therapy in patients with advanced or mRCC.

## Methods

### Study population

In this retrospective study, we reviewed the clinical and pathological records of a total of 39 locally advanced RCC or mRCC patients who were administered VEGFR-TKIs or mTORs between May 2012 and May 2014 at Hirosaki University. We focused on 18 patients who underwent first-line therapy with axitinib. Eligible patients had histologically confirmed clear cell RCC, with local progression or distant metastases. Data on patient demographics and tumor characteristics were obtained from the patients’ medical charts. Memorial Sloan-Kettering Cancer Center (MSKCC) criteria were evaluated based on the five risk factors: low Karnofsky performance status (<80), high LDH (>1.5 times the upper limit of normal), low serum hemoglobin, high corrected serum calcium (>10 mg/dL), and time from initial diagnosis to axitinib treatment of <1 year [3].

The study protocol and informed consent documents were reviewed and approved by the Hirosaki University institutional review board.

### Treatment

Axitinib was administered orally at a dose of 10 mg daily. The axitinib dose was reduced in patients with grade 3 AEs based on the Common Terminology Criteria for Adverse Events (version 4) or two readings of systolic blood pressure at 150 mmHg or higher, or diastolic blood pressure at 100 mmHg or higher, while maintaining maximal antihypertensive therapy. In this study, none of the patients received axitinib dose titration.

### Patient evaluation

Based on the results of percutaneous ultrasonography-guided biopsy, the diagnosis of RCC was confirmed by a single pathologist at our institution.

Baseline evaluations included complete history-taking and physical examinations, assessment of Eastern Cooperative Oncology Group performance status (ECOG PS), abdominal and pelvic computed tomography (CT) or magnetic resonance imaging (MRI), and chest radiography or CT. Tumors were measured at baseline before the administration of axitinib. The response to treatment was assessed using the Response Evaluation Criteria in Solid Tumors, version 1.1 [15]. Bone lesions were considered non-measurable.

All tumors were staged according to the cancer staging manual (7th edition), published by the American Joint Committee on Cancer [16].

### Endpoints and statistical analysis

The primary endpoint was the PFS. The secondary endpoints were overall response rate (ORR) and AEs. The PFS was defined as the time between the initiation of axitinib treatment and the date on the CT scan that identified progressive disease (PD), on other records of clear clinical evidence of PD, or death.

Data were analyzed using IBM SPSS Statistics 20 (IBM Corp., Armonk, NY, USA). Survival after axitinib administration was estimated using the Kaplan–Meier method. All *P* values were 2-sided, and the significance level was set at a *P* value of < 0.05.

## Results

### Patient characteristics and treatment

The pretreatment characteristics of the patients are listed in Table 1. All patients had histologically proven clear cell RCC. The median duration of the administration of axitinib was 10.8 months. Seven patients received reduced axitinib dosing. Five patients received a continuous reduced dose of 3 mg twice daily; of these patients, four had exhibited systolic blood pressure of 150 mmHg or higher, one had suffered general malaise, and one had developed grade 3 proteinuria. Two patients received a continuous reduced dose of 1 mg twice daily due to general malaise.

**Table 1:** **Patient characteristics**

**Variable**	**Value**
Age (years), median (IQR)	73 (64–78)
Sex, number (%)	
Male	12 (67)
Female	6 (33)
ECOG PS, number (%)	
0	14 (77.8)
1	2 (11.1)
2	1 (5.6)
3	1 (5.6)
MSKCC risk group*, number (%)	
Favorable	10 (55.6)
Intermediate	5 (27.8)
Poor	3 (16.7)
Site of metastasis, number (%)	
None	5 (27.8)
Lung	5 (27.8)
Lymph node	4 (22.2)
IVC thrombus	4 (22.2)
Bone	2 (11.1)
Liver	1 (5.6)
Prior to nephrectomy, number (%)	4 (22.2)
Follow-up period (months), median (IQR)	11.5 (5.1–17.4)

* Risk groups are stratified in accordance with the Memorial Sloan-Kettering Cancer Center (MSKCC) criteria associated with shorter survival based on five risk factors: low Karnofsky performance status (<80%), high LDH (>1.5 times the upper limit of normal), low serum hemoglobin, high corrected serum calcium (>10 mg/dL), and interval of <1 year between initial diagnosis and axitinib treatment [3]

ECOG PS, Eastern Cooperative Oncology Group performance status; IVC, inferior vena cava

### Clinical response and PFS

**Figure 1:**
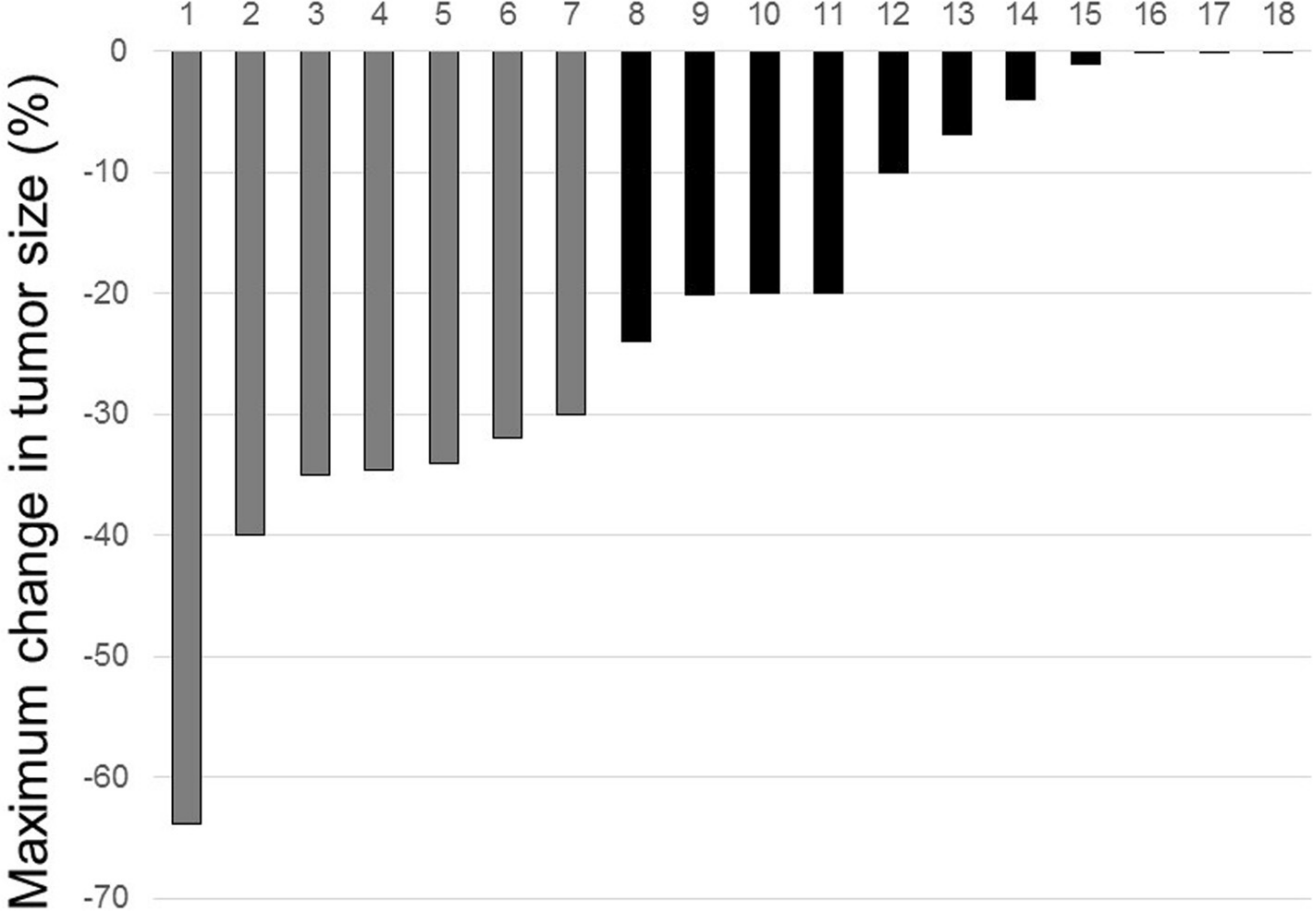
Waterfall plot showing tumor response to axitinib by RECIST. Bars represent individual evaluable patients. Gray, partial response; black, stable disease.

According to the response evaluation criteria in solid tumors (RECIST) criteria, five patients achieved a partial response, nine had stable disease, and four had PD. Median duration of response was 10.8 months (interquartile range [IQR], 5.6-18.3). Tumor shrinkage was observed in 15 patients (primary renal tumor in 10 patients and metastatic site in 5 patients), with a median decrease of 20% in tumor size (IQR, 4.7–33.5; Figure 1).

Three patients underwent open radical nephrectomy after axitinib treatment. Median operative time was 100 minutes (range: 95–128); the median estimated blood loss was 225 mL (range: 50–380 mL). No intraoperative or postoperative complications, as defined by the Clavien-Dindo classification [17], were encountered. The kidney was noted to be adherent to surrounding tissues, including the peritoneum, in all three cases. Pathological examination on final nephrectomy specimens confirmed the presence of clear cell RCC in all three patients; pathological stages were further diagnosed as pT1b, pT3a, and pT3b.

**Figure 2:**
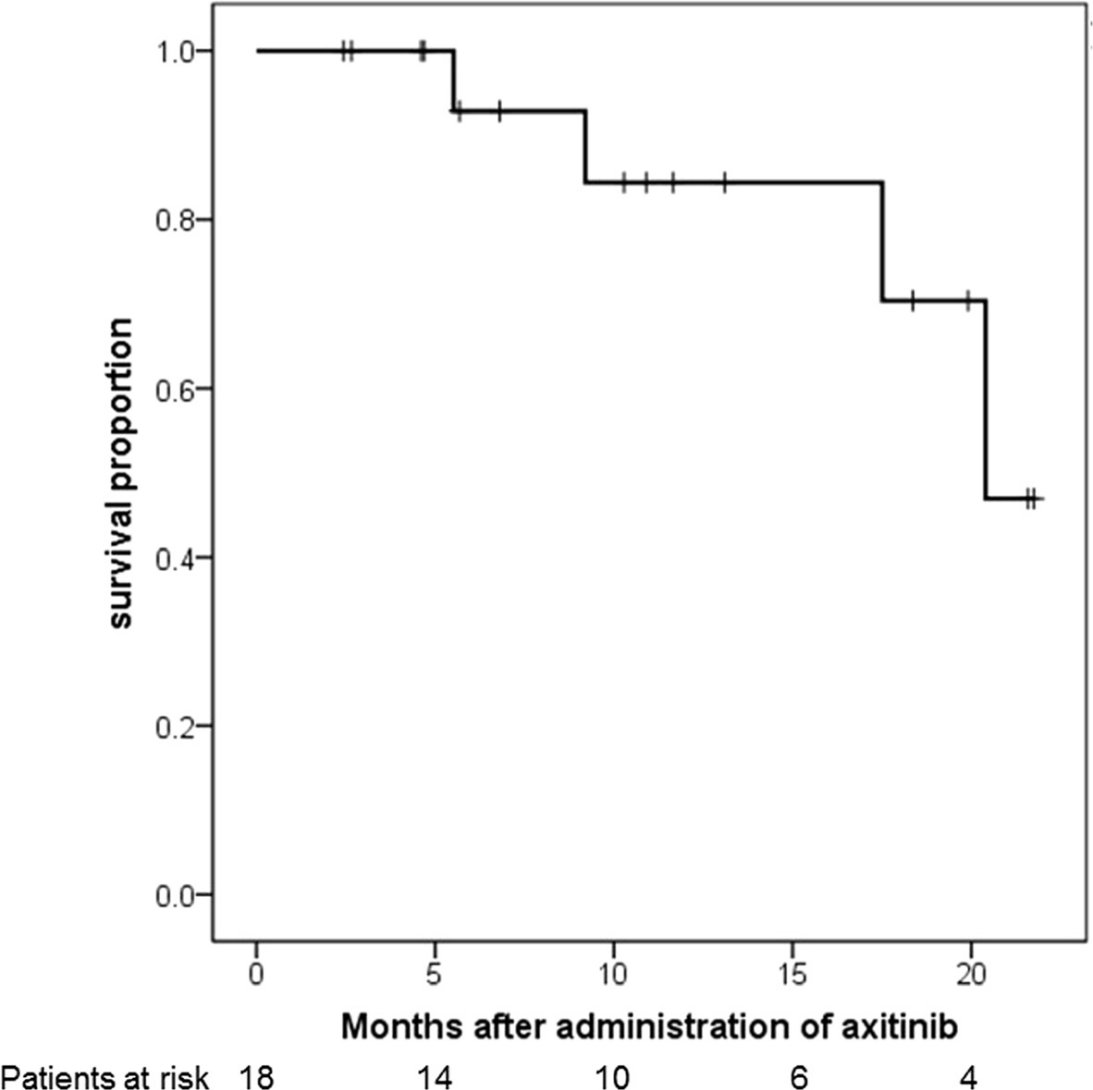
Kaplan-Meier analysis of progression-free survival. The 1-year progression-free survival rate was 84.4% (95% confidence interval, 15.8-21.5).

At the end of the follow-up period, none of the patients had died of cancer or other causes. The 1-year PFS rate was 84.4% (Figure 2). The median PFS was 20.4 months (95% confidence interval, 15.8–21.5). The 1-year PFS rate was 55.6% in the patients with locally advanced RCC (locally advanced group) and 100% in the patients with metastasis (metastasis group) (*P* = 0.373). The median PFS was not reached in the locally advanced group, and it was 20.4 months in the metastasis group. According to the MSKCC risk stratification, the PFS did not differ significantly among all risk groups (*P* = 0.985). Two patients received sunitinib as a second-line treatment, and one patient underwent third-line therapy with pazopanib. One patient received best supportive care. The duration of effectiveness in the patients who were administered sunitinib or pazopanib as second-line treatment were 3 and 6 months, respectively.

### Safety of axitinib treatment

The AEs are shown in Table 2. Hypertension was the most frequent AE. Grade 3 proteinuria was observed in two patients. Other toxicities were infrequent and mild. No serious grade 4 AEs were reported during the study, and there were no toxicity-related deaths.

**Table 2:** **Adverse events**

**Adverse events**	**Any grade, number (%)**	**Grade 3, number (%)**
Hypertension	7 (38.9)	1 (5.6)
Proteinuria	5 (27.8)	2 (11.1)
Hypothyroidism	3 (16.7)	0
General malaise	3 (16.7)	0
Hand-foot syndrome	2 (11.1)	0
Anemia	1 (5.6)	0
Stomatitis	1 (5.6)	0
Renal impairment	1 (5.6)	0

## Discussion

Till 2005, the standard of care was limited to cytokine therapy, including interferon-alpha (IFN-α) and/or interleukin-2, and these treatments were frequently associated with limited efficacy and high toxicity [18]. A better understanding of the molecular mechanisms that target angiogenesis by direct inhibition of VEGF or mTOR has led to improved treatment options for RCC. Clinical trials using novel targeted agents, including TKIs or mTORs, have been evaluated in large randomized controlled studies conducted in both the first- and second-line setting [19]. Of these, sunitinib demonstrated superior efficacy to IFN-α as first-line mRCC therapy, with a median PFS of 11 versus 5 months (*P* < 0.001), respectively, in a randomized phase III trial [4]. Sunitinib is currently regarded as the reference standard of care for the first-line treatment of mRCC.

Several studies have reported widely variable rates and grades of sunitinib-related AEs [6,8,9,20]. The rates of incidence for the most common grade 3/4 AEs that require dose discontinuation and/or reduction, including hand-foot syndrome (HFS), stomatitis, and hypertension were similar to the rates reported in previous trials [21,22]. However, differences in ethnicity-based treatment tolerance may have also played a role. Miyake et al. reported that the rates of incidence of AEs ≥ grade 3 in a phase III clinical trial and a phase II Japanese clinical trial were 61% and 95%, respectively [9]. Similarly, *ad hoc* analyses indicate that several AEs occur at a significantly higher rate in Asian patients relative to Caucasian patients; for example, HFS occurred in 70% of Asian patients compared with 28% of Caucasian patients (P < 0.001) [8]. Although the standard sunitinib schedule involves four weeks of treatment and two weeks of rest, a modified schedule of sunitinib treatment, with two weeks of treatment and one week of rest, was associated with significantly decreased toxicity [23].

Common AEs of axitinib include diarrhea, hypertension, fatigue, anorexia and weight loss. The safety profile of axitinib is generally manageable with standard medical intervention [24]. In the AXIS study, discontinuation rates due to treatment-related AEs were 4% in the axitinib arm and 8% in the sorafenib arm, while dose interruptions and reductions were required in 77% and 31% of axitinib recipients [25]. The AXIS study protocol allowed for dose escalation in the absence of hypertension or grade 2 AEs, which may have been partially responsible for the subsequent increase in dose-reduction rate [26].

In this study, the treatment-naïve cRCC patients had a relatively longer PFS without axitinib dose titration, compared with other clinical trials. Rini et al. reported that in treatment-naïve mRCC patients who initially tolerated axitinib at a dose of 5 mg twice daily, a significantly higher proportion achieved an objective response with axitinib dose titration than with placebo titration [27]. Furthermore, based on the results from a phase 3 trial evaluating axitinib versus sorafenib in treatment-naïve patients with mRCC, the median PFS was 10.1 months with axitinib and 6.5 months with sorafenib (*P* = 0.038) [13]. In addition, median PFS was 13.7 months with axitinib and 6.6 months with sorafenib in patients with ECOG PS 0 (*P* = 0.022) [13]. In this study, the differences in PFS were not significant between all risk groups, according to the MSKCC risk stratification. Although brief exposure to higher axitinib doses may achieve immediate tumor shrinkage, a substantial proportion of patients may subsequently be forced to lower axitinib doses, which may lead to lower rates of long term disease control.

The current study has several limitations. First, it is a retrospective study, with an inherent potential for bias. Second, a relatively small number of patients were enrolled in this study, and the follow-up period was relatively short. In this study, the number of enrolled patients was relatively high age compared with other randomized trials [13,27]. AEs were also effectively managed with medications or axitinib dose reduction in this study. Although a large proportion of patients in other randomized control studies were recruited from North America and Western Europe, some patients were recruited from Asia, but the number was not large enough. Therefore, axitinib as first-line therapy may provide a treatment option for selected Japanese patients with locally advanced or mRCC.

## Conclusions

In the current study, axitinib showed improved oncological outcomes and an acceptable safety profile as the first-line therapy for advanced RCC or mRCC in treatment-naïve patients. Thus, first-line therapy with axitinib may provide a promising treatment option for advanced RCC or mRCC patients. Further trials in the first-line setting are warranted.

### Consent

Written informed consent was obtained from the patient for publication of this case report and the accompanying images. A copy of the written consent is available for review by the Editor-in-Chief of this journal.

## Abbreviations

AEs: Adverse events
cRCC: clear cell renal cell carcinoma
CT: Computed tomography
ECOG PS: Eastern Cooperative Oncology Group performance status
IFN-α: Interferon-alpha
mRCC: metastatic renal cell carcinoma
mTORs: mammalian target of rapamycin inhibitors
MRI: Magnetic resonance imaging
MSKCC: Memorial Sloan-Kettering Cancer Center
ORR: Overall response rate
PD: Progressive disease
PFS: Progression-free survival
RCC: Renal cell carcinoma
RECIST: Response evaluation criteria in solid tumors
TKIs: Tyrosine kinase inhibitors
VEGF: Vascular endothelial growth factor
